# Determining future aflatoxin contamination risk scenarios for corn in Southern Georgia, USA using spatio-temporal modelling and future climate simulations

**DOI:** 10.1038/s41598-021-92557-6

**Published:** 2021-06-29

**Authors:** Ruth Kerry, Ben Ingram, Esther Garcia-Cela, Naresh Magan, Brenda V. Ortiz, Brian Scully

**Affiliations:** 1 Department of Geography, Brigham Young University, Provo, UT USA; 2 Facultad de Ingeniería, Universidad de Talca, Talca, Chile; 3 Clinical Pharmaceutical and Biological Sciences, University of Hertfordshire, Hertfordshire, UK; 4 Applied Mycology Group, Cranfield University, Cranfield, UK; 5 Crop, Soil, and Environmental Sciences Department, Auburn University, Auburn, AL USA; 6 U.S. Horticultural Research Laboratory, Fort Pierce, FL USA

**Keywords:** Climate change, Environmental impact, Agroecology, Biogeography, Climate-change ecology

## Abstract

Aflatoxins (AFs) are produced by fungi in crops and can cause liver cancer. Permitted levels are legislated and batches of grain are rejected based on average concentrations. Corn grown in Southern Georgia (GA), USA, which experiences drought during the mid-silk growth period in June, is particularly susceptible to infection by *Aspergillus* section *Flavi* species which produce AFs. Previous studies showed strong association between AFs and June weather. Risk factors were developed: June maximum temperatures > 33 °C and June rainfall < 50 mm, the 30-year normals for the region. Future climate data were estimated for each year (2000–2100) and county in southern GA using the RCP 4.5 and RCP 8.5 emissions scenarios. The number of counties with June maximum temperatures > 33 °C and rainfall < 50 mm increased and then plateaued for both emissions scenarios. The percentage of years thresholds were exceeded was greater for RCP 8.5 than RCP 4.5. The spatial distribution of high-risk counties changed over time. Results suggest corn growth distribution should be changed or adaptation strategies employed like planting resistant varieties, irrigating and planting earlier. There were significantly more counties exceeding thresholds in 2010–2040 compared to 2000–2030 suggesting that adaptation strategies should be employed as soon as possible.

## Introduction

Climate change threatens future food security due to the probable increase in temperature and changes in precipitation patterns which could affect the distribution of where different crops may be viably grown^1,2^. Land suitability and capability classifications are used to evaluate the best growing areas for specific crops and to identify factors that might limit their growth^3,4^. Key factors in determining these classifications include fluctuations in moisture availability and temperature regimes. Land suitability and capability classifications for particular crops in a given region with or without irrigation need recalculating to take account of future climate change scenarios. In addition to such classifications that are based on the optimum growing conditions for a given crop, increased susceptibility to diseases from bacteria or fungal pathogens are limiting factors which need to be considered. Of particular importance in corn is infection by members of the *Aspergillus* section *Flavi* during the silking period (June in Georgia, USA) and concomitant contamination of the cobs with AFs^5–8^.

AFs, especially aflatoxin B1 (AFB1) are class 1a carcinogens which cause liver cancer in humans and animals^9–11^. The key species responsible for AFs contamination in corn are *Aspergillus flavus* and sometimes *A. parasiticus*, which are able to colonize ripening cobs, especially via pest damage at 30-35 °C during the milky ripe to dough ripeness stages of the kernels. This is especially prevalent when the corn plants are under drought stress^8,12–14^. Thus, interacting climatic conditions in some years of higher than normal maximum temperatures and lower than normal rainfall represent conditions conducive to AFs contamination^15,16^. Because of the extreme toxicity of AFB1 and AFs generally, many countries have strict legislative limits on the maximum permitted concentrations of these two categories of toxins in cereals and other foodstuffs for human or animal consumption^17,18^. The United States Food and Drug Administration (FDA) has a limit of 20 ppb total AFs in corn, peanuts, cottonseed and other feed/feed ingredients intended for animal consumption, particularly for immature animals. The limit is higher (100 ppb) for use of corn and peanut products for beef cattle, pigs and mature poultry^19^. The European Union (EU) generally has the strictest limits of permissible total AFs in various foodstuffs with a maximum of 4–15 ppb^20,21^.

Thus, all batches of grain are sampled for AFs by taking numerous samples from throughout the batch, then mixing the grain and determining the ‘average’ AFs concentration of the batch based on one bulked sample^20^. If the average contamination level of this one sample is above the legislative limits then the whole batch of grain is rejected. The impact of climate-related abiotic factors may result in more stress on corn production in a particular region thus increasing the potential for rejection and impacting the viability of growing corn in a specific area.

Several studies of ripening corn and stored corn have investigated the impact of climate-related abiotic factors on AFs or AFB1 contamination^8,22–24^. These suggest that elevated temperatures (+4 to 5 °C above optimum), drought stress and exposure to increased CO_2_ levels (400 vs 1000 ppm) resulted in an increase in contamination with AFB1. Summer corn crops are very prone to AFs contamination in the Southern USA^25^ due to high temperatures, rainfall variability, light textured soils and lack of irrigation infrastructure^6^. All these factors compound crop water stress and subsequent remedial actions often do not alleviate the impacts on yield or contamination with these toxins. In Southern GA, the temperature and rainfall conditions in the critical month of June, which corresponds to the fragile mid-silk growth stage of corn growth, have been linked to the risk of AFs contamination^26,27^. Salvacion et al.^27^ evaluated the influence of weather variables in different growth stages based on exceedance of the FDA 20 ppb threshold and it was found that drought conditions in the month of June were by far the most significant. Based on this finding, Kerry et al.^15^ used thresholds in June maximum temperatures (TMax) and June rainfall (RF) above and below climate normals, respectively to identify years and counties with different levels of contamination risk. Their approach was validated by comparing the AFs concentrations for the different risk zones which were found to be significantly different and reflected the order of risk identified. They also found a strong relationship (r=0.802) between the percentage of counties exceeding 2 weather risk factor thresholds (June RF less than, and June TMax greater than 30-year normals) and the percentage of counties with > 20 ppb AFs. This approach was further validated by the study of Yoo et al.^16^ where profile regression was used to identify high and low risk clusters for AFs contamination and which factors were the most influential. June TMax and June RF were consistently found to be important. In addition, in another study, weather variables were shown to explain 50-76% of the variation in AFs concentrations^28^.

Based on the approach of Kerry et al.^15^, Navarro et al.^29^ developed a decision support tool that could be useful to extension services to help determine the counties at greatest risk of AFs contamination during a given growing season. Information on the potential contamination risk in a given area could enable farmers to employ various management strategies before the season, during crop development and at harvest. Such strategies include (1) planting resistant varieties or varying seeding rates in high-risk zones at the start of the season, (2) varying irrigation and fungicide applications during the season and (3) at harvest separating grain from zones with different contamination risk prior to storage^16^. In addition, knowing the characteristics of years with different levels of risk could reduce spending on expensive AFs testing surveys in low-risk years and in individual counties.

Battilani et al.^30^ looked at the impacts of temperature changes of 2 °C and 5 °C on model simulated AFs contamination of maize in Europe but they did not investigate the combined probable impact of temperature and rainfall changes. Future climate projections (temperatures and rainfall) can be estimated based on the IPCC (Inter-governmental Panel on Climate Change) greenhouse gas models such as the RCP 4.5 (Representative Concentration Pathway) model. This is an intermediate emissions scenario which is a stabilization scenario assuming that enacted policies will cause stabilization in carbon emissions by 2100^31^.

The RCP 8.5 model is a high emissions scenario that assumes what is frequently described as a “business as usual” with no enacted policies to cut greenhouse gases. It is hoped that the RCP 4.5 scenario is a more realistic outcome compared to the RCP 8.5 scenario, however, a 2020 study^32^ found that historical total cumulative CO_2_ emissions are within 1% of those in the RCP8.5 model and it is also the best match to mid-century under current and stated policies**.** The IPCC climate change summary^33^ suggests that under the RCP 4.5 scenario mean increases in mean global surface temperature compared with the 1986–2005 reference period would be 1.4 °C and 1.8 °C for the 2046–2065 and 2081–2100 periods, respectively. Under the RCP 8.5 scenario mean changes in mean global surface temperature compared with the 1986–2005 reference period would be 2.0 °C and 3.7 °C for the 2046–2065 and 2081–2100 periods, respectively. The range of temperature increases could, however, be as high as 4.8 °C for the RCP 8.5 scenario^33^. The RCP 4.5 and RCP 8.5 emissions scenarios were used to predict June TMax and June RF for each county in southern GA for 30-year periods from 2000 to 2100. The aim of this study was to explore how climate change abiotic factors could affect patterns of AFs risk, both spatially and temporally in southern GA, USA under the RCP 4.5 and RCP 8.5 scenarios compared to the 1977–2004 county level AFs and weather survey. The thresholds for June RF and June TMax developed by Kerry et al.^15^ to define high and low risk years and counties were used in this process. It is recognized that in the 1977–2004 southern GA AFs survey there were other factors that could influence AFs concentrations such as soil type, planting and harvest dates. However, several studies suggest that weather factors have the most influence on AFs concentrations^15,16,28^. The purpose of this investigation of future AFs contamination risk was to help inform whether the location of zones where corn is grown in southern GA may need to be changed due to climate change or if management practices need to be adjusted to minimise the levels of these toxins in this staple commodity.

## Results

### Past patterns of corn growth, weather and aflatoxin contamination

**Figure 1:**
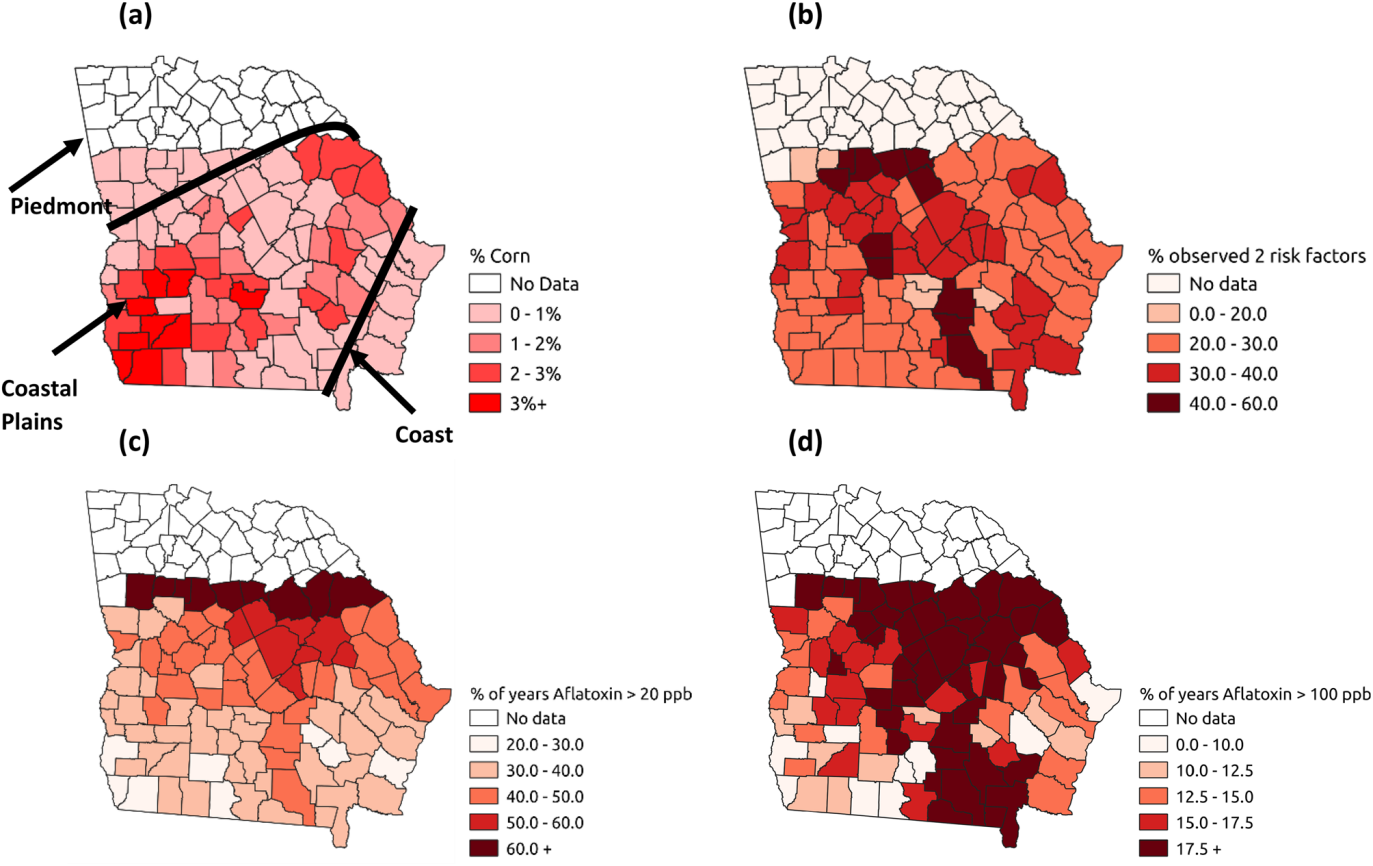
County maps of Southern Georgia, USA showing: **(a)** % Corn growing area and physiographic regions, **(b)** % observed two weather risk factor thresholds exceeded 1980–2004, **(c)** % years with > 20 ppb Aflatoxins, **(d)** % years with > 100 ppb Aflatoxins.

Figure 1a shows the percentage area currently dedicated to corn crops in southern GA. Counties in the south-west and north-east have the highest density of corn crops and the central and north-western counties the lowest density of corn crops. The vast majority of corn within the state of GA is grown in the southern coastal plains area. The boundary between this area and the piedmont area directly north of it runs diagonally from the south west of the region to the north eastern area of southern GA. A second and much smaller corn production region exists in the north and north-western quadrants of the state (not shown in Fig. 1a), but corn grown in this region is typically lower yielding and less susceptible to AFs contamination, due to higher elevation and cooler summer temperatures. Thus, this region was not sampled in the present study. Figure 1b shows the percentage of years each county had two weather risk factors (June TMax > 30 °C and June RF < 50 mm) for historical (1977–2004) climate data. The percentage of years with two weather risk factors is greatest across the central parts of southern GA along a north-west to south-east trajectory. Figure 1c,d show the Poisson kriged percentage of years that historical (1977–2004) AFs concentrations were greater than the FDA 20 ppb and 100 ppb limits, respectively. The northern and central counties in a roughly north–south trajectory have the largest percentages of years with AFs concentrations greater than both 20 ppb and 100 ppb. These central counties are where least corn is grown (Fig. 1a), show the greatest weather risk of AFs contamination (Fig. 1b) and were the most contaminated when surveyed (Fig. 1c,d). This suggests that in terms of land suitability, more frequent drought conditions in these counties limited the ability to produce corn without AFs contamination.

### Future weather patterns and risk of aflatoxin contamination—temporal analysis

**Figure 2:**
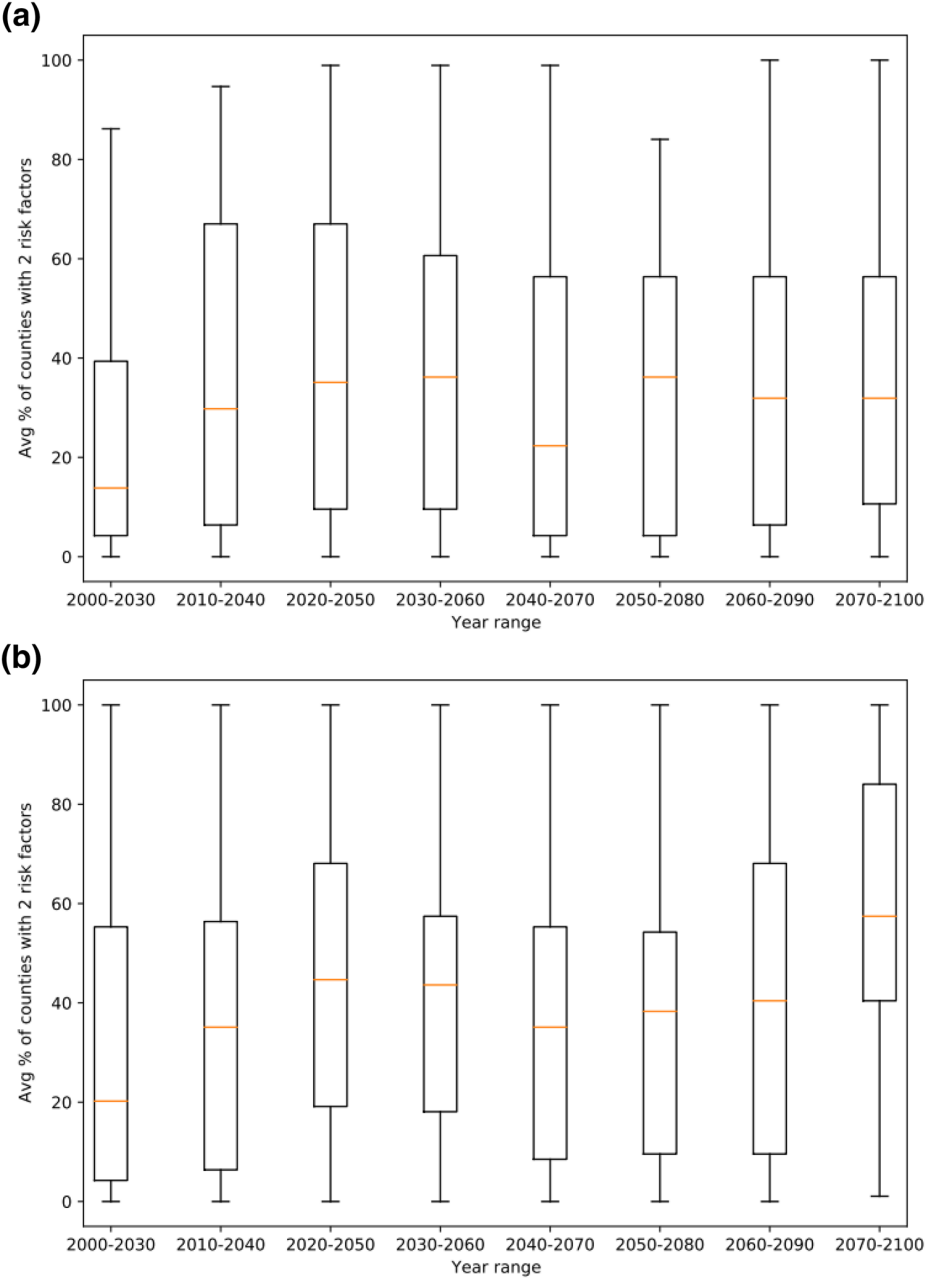
Average percentage (%) of counties in southern Georgia, USA predicted to exceed two weather risk factor thresholds for 30 years periods under the **(a)** RCP 4.5 scenario and **(b)** RCP 8.5 scenario.

Future climate projections for June TMax and RF were estimated assuming both the RCP 4.5 and RCP 8.5 emissions scenarios for each year from 2000 until 2100. Risk factors (June Tmax > 30 °C and June RF < 50 mm) were calculated and analysed for 30-year periods until 2100. The percentage of counties which exceeded both risk factor thresholds (June Tmax > 33 °C and June RF < 50 mm) was calculated for each year. For each 30-year period, box and whisker plots were generated to show the distributions of the proportion of counties exceeding thresholds in each year (Fig. 2). The RCP 4.5 (Fig. 2a) and RCP 8.5 (Fig. 2b) emissions scenarios both show increases over time in the median percentage of counties with two weather risk factor thresholds being exceeded. However, both scenarios show a plateauing in these increases and the RCP 4.5 shows a marked decrease for the 2040–2070 period. Both scenarios show a peak in the median percentage for the 2030–2060 period. However, for the RCP 8.5 scenario following some stabilization, there is a marked increase for the 2070–2100 period. Figure 2 also shows that the mean percentage for the RCP 8.5 scenario is slightly higher than for the RCP 4.5 scenario. In addition, for the RCP 8.5 scenario, the distribution is slightly more dispersed and skewed towards high levels as in most box and whisker plots, the median line is located in the top half of the box (Fig. 2b). The data for each county of the percentage of years in each 30-year period that two weather risk thresholds were exceeded was used in Mann Whitney U comparison tests. Each 30-year period was compared with the 2000–2030 data under the same emissions scenario. For each time period under the RCP 4.5 scenario, there was a significant (p < 0.020) difference between it and the 2000–2030 period. For each 30-year time period under the RCP 4.5 scenario, the percentage of counties exceeding two weather risk factor thresholds was greater than that for 2000–2030. The difference was least significant for the 2050–2080 period (p = 0.017). For each time period under the RCP 8.5 scenario, there was a significant (p < 0.010) difference between it and the 2000–2030 period apart from for the 2040–2070 period (p = 0.048). For each 30-year time period under the RCP 8.5 scenario, the percentage of counties exceeding two weather risk factor thresholds was greater than that for 2000–2030. The difference was least significant for the 2050–2080 period (p = 0.017).

**Figure 3:**
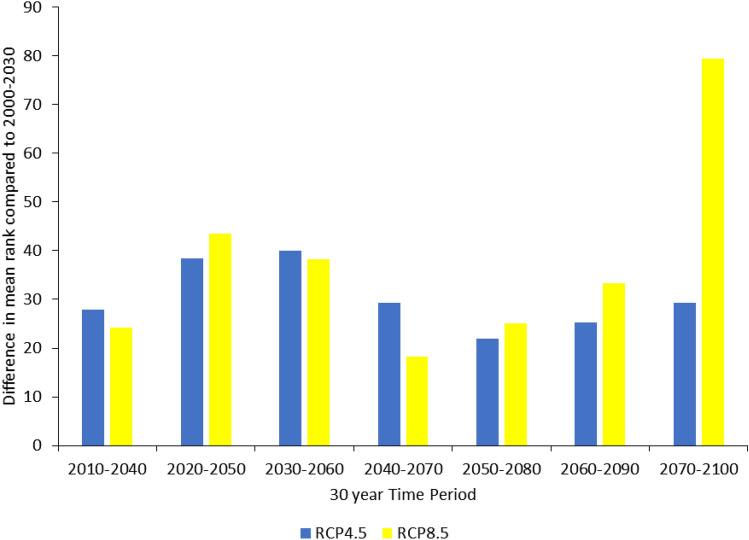
Bar chart showing the difference in mean ranks for Mann Whitney U tests comparing the 2000–2030 and different time periods for RCP4.5 and RCP8.5.

Figure 3 shows the difference between mean ranks for counties in each time period compared with the 2000–2030 period. This shows that the difference in mean ranks is similar under both emissions scenarios in the first three time periods. Figure 3 also shows that in the 2070–2100 time period, there is a very big difference in the proportions of counties with two risk factors compared to 2000–2030 for the RCP 8.5 scenario.

**Figure 4:**
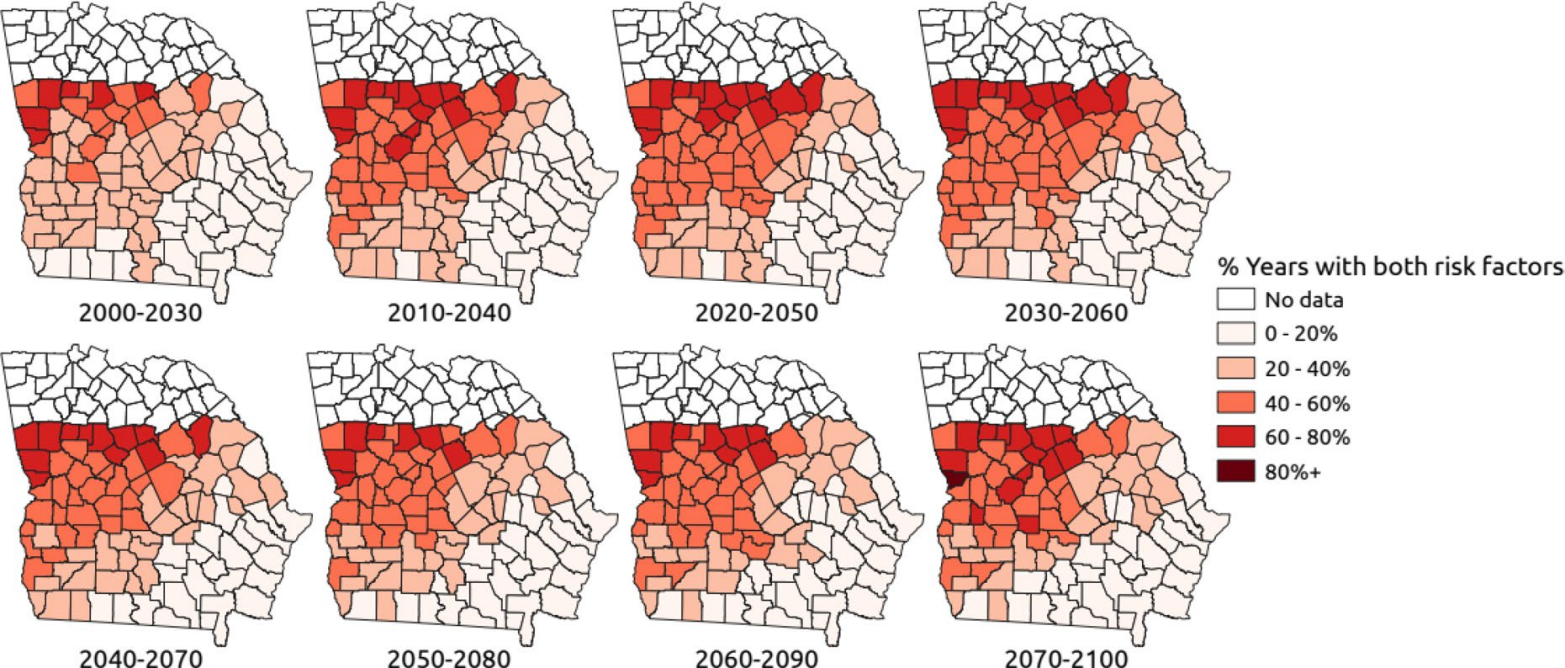
County maps of southern Georgia, USA, with the percentage of years predicted to exceed 2 weather risk factor thresholds assuming the RCP 4.5 scenario.

**Figure 5:**
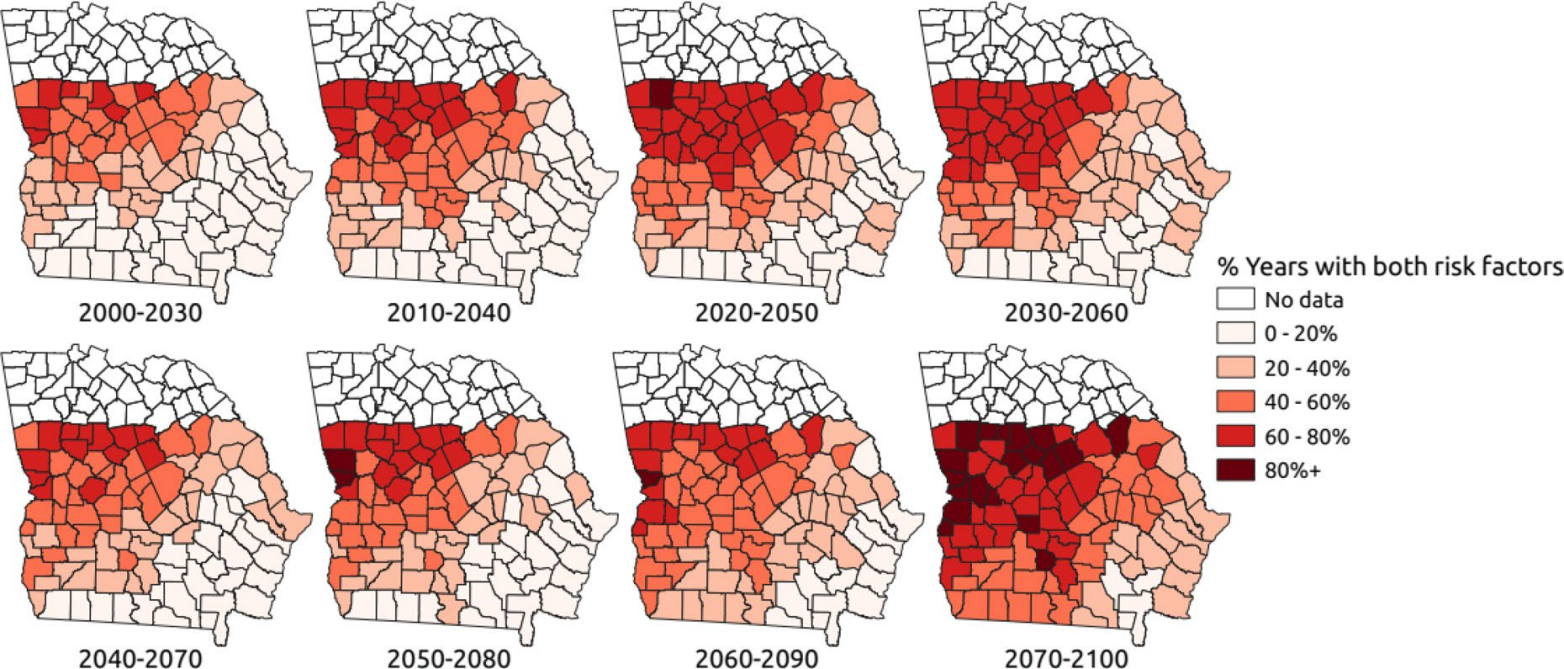
County maps of southern Georgia, USA, with the percentage of years predicted to exceed two weather risk factor thresholds assuming the RCP 8.5 scenario.

Tables 1 and 2 show the numbers of counties (Figs. 4 and 5) with different percentages of years having 2 weather risk factors. For each time period, the percentage with the largest number of counties in it is indicated by a *. For the RCP 4.5 emissions scenario (see Table 1), the dominant proportion of years with two weather risk factors in 2000–2030 is 20–40%. For the next three time periods, the dominant proportion increases to 40–60%. For the last two time periods the dominant percentage is 0–20%. However, the number of counties with 60–80% of years with 2 weather risk factors is larger than 2000–2030 for all other time periods. For the RCP 8.5 emissions scenario (see Table 2), the dominant proportion of years from 2000 to 2030 with two weather risk factors is 0–20%. This is low in comparison to Fig. 1b from the 1977–2004 survey, where over half of the counties have two weather risk factors 20–30% of the time. For all time periods, the RCP 8.5 scenario has larger numbers of counties with 60–80% of years exceeding 2 weather risk factors and for the 2070–2100 period 19 counties have 2 weather risk factors in > 80% of years (Table 2).

**Table 1:** Number of counties (n = 94) with different percentages of years with 2 weather risk factor thresholds exceeded under the RCP 4.5 scenario.

% of years with 2 risk factors	Number of counties							
	2000–2030	2010–2040	2020–2050	2030–2060	2040–2070	2050–2080	2060–2090	2070–2100
0–20	34	28^a^	25	26	25	29	31^a^	30^a^
20–40	41^a^	25	24	22	30^a^	28	24	24
40–60	13	28^a^	32^a^	33^a^	27	30^a^	30	25
60–80	6	13	13	13	12	7	9	14
80–100	0	0	0	0	0	0	0	1

^a^Dominant proportion of years with 2 risk factors for 30-year period.

**Table 2:** Number of counties (n = 94) with different percentages of years with 2 weather risk factor thresholds exceeded under the RCP 8.5 scenario.

% of years with 2 risk factors	Number of counties							
	2000–2030	2010–2040	2020–2050	2030–2060	2040–2070	2050–2080	2060–2090	2070–2100
0–20	41^a^	31	21	19	27	27^a^	14	3
20–40	24	20	23	29^a^	28^a^	27^a^	28	15
40–60	22	25^a^	21	17	26	23	35^a^	29^a^
60–80	7	18	28^a^	29^a^	13	15	16	28
80–100	0	0	1	0	0	2	1	19

^a^Dominant proportion of years with 2 risk factors for 30-year period.

### Spatial analysis

In each 30-year time period, both climate risk factors (June TMax > 30 °C and June RF < 50 mm) were calculated for each year in each county. The percentage of years which exceeded both risk factor thresholds was calculated for each county and plotted as maps for the RCP 4.5 and RCP 8.5 emissions scenarios, respectively (Figs. 4 and 5). Figure 4 shows that the greatest increase in AFs contamination risk over time is in the north west of the study region, but several counties in the south-western part of the region are also predicted to exceed thresholds in 20–60% of years rather than 0–40% of years as in 2000–2030. Spatially, it is interesting to note that the weather risk is increasing most in western and northern parts of the region (Fig. 4). As the temporal data suggested, the risk of AFs contamination under the RCP 8.5 scenario is generally more severe. Risk is greatest in the north west of the region with many counties in this area predicted to experience two weather risk factors in 60–80% of years in the 2020–2050, 2030–2060 and 2070–2100 periods. The number of counties in the south west of the region, currently the dominant corn growing area (Fig. 1a), which have > 40% of years with two weather risk factors increases from the 2020–2050 period onwards and is particularly large in the 2060–2090 and 2070–2100 periods. The number of counties with < 40% of years with two weather risk factors is small for the 2070–2100 period.

## Discussion

Over time, particularly for the RCP 8.5 scenario, the main corn growing counties are increasingly at risk of AFs contamination and exceed two weather risk factors in increasing proportions of years. The spatial patterns for the RCP 4.5 and 8.5 scenarios also suggest a shift away from the central counties in southern GA being at the highest risk and a move towards the north west and south-western counties being at greater risk from toxin contamination. The south-western counties are currently (Fig. 1a) the dominant corn growing areas in southern GA. There are many uncertainties associated with the future climate predictions under both the RCP 4.5 and 8.5 scenarios as evidenced by the large spread in the distribution of the box and whisker plots. The whiskers for most time periods extend from 0 to 100% and the boxes are 40–60% in height. The exact process of downscaling of weather data to the county level and monthly data used in the Climate toolbox (https://climatetoolbox.org/tool/future-time-series) needs further investigation and it would be useful to have a more up to date county-level AFs survey for southern GA to see if patterns in actual contamination/risk have changed since the 1977–2004 period as the projected climate data for 2000–2030 suggest they have.

The modelling approach here only takes account of June TMax and RF which were the most important factors linked to AFs concentrations for the 1977–2004 survey. There are, however, several other factors that can influence AFs concentrations such as soil type, warmer temperatures earlier and later in the growing season, and this model assumes that these factors will continue to have less influence in the future, although they may not. Another issue is the hard thresholds used for the risk factors. This approach works well when looking at a large number of counties and years as the general patterns can be observed which show the level of uncertainty in the risk. However, farmers should avoid applying these hard thresholds to individual years and locations. Rather they should consider how close to each threshold the weather in the given location is and if TMax and RF are both close to thresholds but do not exceed them, farmers can consider their location as high risk in a particular year. Further research should look at quantifying risk of AFs threshold exceedance as a function of June Tmax and RF rather than hard thresholds. Also, more risk factors could be included in the estimation of future AFs contamination risk to reduce uncertainty in risk estimates.

**Figure 6:**
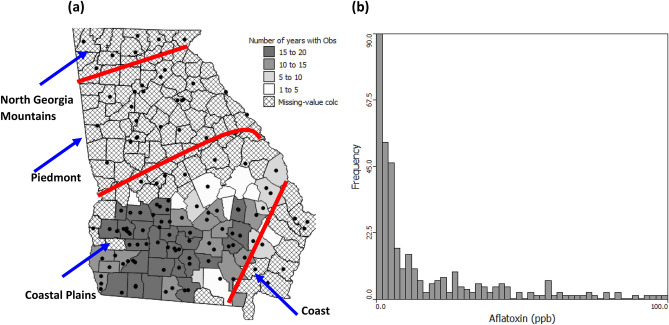
**(a)** Map showing the number of years with Aflatoxins observations in each county and the location of weather stations (black dots) and physiographic zones within Georgia separated by red lines and **(b)** Histogram of county level Aflatoxins values < 100 ppb for southern Georgia, USA, 1977–2004, 61 observations (not shown) were between 100 and 4807 ppb.

In spite of the uncertainties and potential loss of accuracy due to discretization in the modelling approaches used here, the spatial and temporal patterns are clear and statistically significant. They suggest a general increase in the number of counties at risk of high levels of AFs contamination over the next 80 years with some stabilization for both emissions scenarios. They also suggest a shift in the spatial patterns of highest risk counties towards those that currently grow most corn. Battilani et al.^30^ had similar results for simulated corn AFs contamination risk in Europe based on temperature-based modelling under increases of average temperature above pre-industrial levels of 2 and 5 °C. They noted that the level of contamination risk would increase overall and that larger areas, northerly areas previously not at risk, would be at greater risk of contamination. It would be interesting to see how their results might change if the impact of climate change on precipitation patterns were also factored into their modelling approach as it was here. The pattern with latitude within Georgia is however, the reverse of that observed by Battilani et al.^30^ with locations further north and west being at greatest risk and this risk decreasing in a south-easterly direction. This pattern shows the ameliorating effect of proximity to the coast (found at the south-eastern edge of the state, Fig. 6) on high temperatures and drought conditions, but also shows that this effect is lessened as mean global temperatures increase. Nevertheless, both pieces of research suggest an increase in general contamination risk and a shift in the locations at risk, which suggests that land capability and suitability classifications may need revising with AFs contamination as a potential limiting factor. Farmers may thus need to refine the zones where they grow corn or alter their management strategies.

Farmers will need guidance from extension services if they are to adapt to future climate change-related abiotic shifts and their likely effect on AFs contamination and subsequent rejection of batches of corn. Extension services will need to provide timely advice on adaptation strategies as the percentage of times counties exceeded two weather risk factors in the 2010–2040 period was found to be significantly greater than the 2000–2030 period for both the RCP 4.5 and 8.5 scenarios. However, the effects of climate change are less severe for the RCP4.5 scenario. Within the state of GA, the predicted patterns of future weather risk for AFs contamination suggest that farmers should start to grow more corn in the central and south eastern parts of the region, but not near the coast where soils are very sandy and require irrigation. If corn distribution patterns are not altered, farmers in the southern GA region should improve and invest in irrigation infrastructure, plant crops earlier and use cultivars that are more resistant to *A. flavus* infection and have some drought tolerance. They will probably also need to increase their fungicide budget. Land suitability and capability classification for different crops should be recalculated in general to take into account such climate change scenarios and perhaps future land suitability classifications could be developed to incorporate AFs contamination risk as a possible limiting factor to growing corn and other crops.

## Methods

### Aflatoxin data

A 1977–2004 AFs survey was used in several studies to investigate the factors important to AFs contamination^15,16,26,27^ and develop a risk factor approach based on June weather data. County-level AFs data were collected between 1977 and 2004 in 53 counties in southern GA, USA (Fig. 6a). The data were collected for different numbers of the 53 counties each year. The year with samples for the minimum number of counties was 1990 with just 23 counties being sampled. The year with most counties sampled was 1978 with 45 counties being sampled. Corn grain samples were collected at harvest using a grab sampling technique where 10 ears were collected within a randomly selected field for each sampling and there was an average of 3 replications for different fields per county. Corn samples were ground immediately after harvest and stored in a cold room to prevent further AFs build-up in the short period of time between sample collection and analysis. There is some uncertainty in the AFs measurements (ppb) as there was a change in the laboratory methods that were used during the survey period. Thin layer chromatography was used until the late 1990s, subsequently the VICAM AflaTest^34^ was used in more recent years. AFs measurements were made by the USDA-ARS Crop Protection and Management Research Unit and the University of Georgia, Natural Products Laboratory in Tifton, GA.

As AFs data were not available for every county each year, geostatistical methods were used to estimate missing values and fill these data gaps. The histogram of the data for the whole survey period (n = 818) approximates a Poisson distribution (Fig. 6b) with lots of low values and a long tail with a small number of high values. Typical method of moment variograms become erratic in the presence of highly skewed data^35^ and when there are too few data for individual years (< 100) to compute a reliable variogram for each year^36^. To circumvent these problems and to provide a good temporal summary for comparison with climate rather than weather patterns in individual years, Poisson kriging of rate variables calculated for the whole survey period was performed. The proportions of years with AFs concentrations greater than the FDA 20 ppb and 100 ppb thresholds were calculated and Poisson kriged^37^ to the centroid of each county. The FDA also has 200 ppb and 300 ppb thresholds for corn feeds, but these are specific to finishing swine > 100 lbs and finishing beef cattle, respectively. The 20 ppb and 100 ppb FDA thresholds were used for this research as they are generally applicable to more animal species. Poisson kriging down-weights the influence of counties where rates are based on fewer years of observations in the variogram computation and the estimation process. For full details of Poisson kriging in general and specifically related to the southern GA AFs survey see Monestiez et al.^37^ and Kerry et al.^15^, respectively.

### Corn growing area

The corn growing area for each county was calculated using the CropScape—Cropland data layer produced by the National Agricultural Statistics Service (NASS, http://nassgeodata.gmu.edu/CropScape/). The first year this was available for southern GA was the 2008/2009 growing season so this was used as the time closest to the survey period (1977–2004). The CropScape data layer identifies 56 m or 30 m pixels where corn was grown for each growing season. Isolated corn pixels were not considered, only pixels that were clustered together with at least 3 connected 56 m^2^ pixels or 9 connected 30 m^2^ pixels were considered so that the proportions of corn grown were not reflecting isolated mis-identified pixels that were not being used for agricultural purposes. Using CropScape data from just one year assumes that the areas growing corn in southern GA have not changed markedly during the study period. Non-spatial data on corn area grown in each county by year were available using the quick stats tool of USDA-NASS https://quickstats.nass.usda.gov/ and suggested that the highest corn-producing counties are consistent in time and are consistent with the proportions of county area growing corn identified by the 2008/2009 CropScape data.

### Weather data for the aflatoxin survey period

Monthly weather data were obtained for each year in the AFs survey period (1977–2004) from the GA Weather Network (http://georgiaweather.net). The locations of weather stations (82 in total) are shown as black dots in Fig. 6a. Based on the findings of several previous studies^15,16,26,27^, June TMax and June RF were the weather data used for each year. Figure 6a shows that there are not weather stations in every county so the June TMax and June RF values for each year were ordinary kriged to county centroids to fill these data gaps with estimates. The 30-year normal for June TMax in southern GA is 33 °C and 50 mm for June RF. The ordinary kriged weather data were used to determine the percentage of years in the 1977–2004 study period that had June TMax > 33 °C and June RF < 50 mm, i.e., higher temperatures and lower rainfall than normal.

### Future climate projections

Future climate projections for June TMax and RF were determined assuming both RCP 4.5 and RCP 8.5 emission scenarios for each variable in the year range 2000 to 2100. Projections were made using the Climate Toolbox^38^. These projections are generated from the IPCCs Coupled Model Inter-comparison Project (CMIP5)^39^ and based on two future scenarios, all with a resolution down-scaled to 4 km for the entire USA. From this down-scaled data, the Climate Toolbox interface gave estimates of June TMax and RF for each county in southern GA. These were then used to calculate weather risk factors (June Tmax > 30 °C and June RF < 50 mm) for all counties for each year. The percentage of counties which exceeded both risk factor thresholds was calculated for each year.

## Data Availability

The datasets generated during and/or analysed during the current study are not publicly available but are available from the corresponding author on reasonable request.
